# Aortic calcium score predicts early outcomes in aortoiliac revascularization

**DOI:** 10.31744/einstein_journal/2025AO0527

**Published:** 2025-03-10

**Authors:** Adalberto Batalha Megale, Nelson Wolosker, Vitoria Magliari Kalil, João Mário Nigro, Carolina Sciammarella Wakisaka, Bianca Oberhuber Dias, Marcelo Passos Teivelis, Marcelo Assis Rocha, Cynthia de Almeida Mendes

**Affiliations:** 1 Hospital Israelita Albert Einstein São Paulo SP Brazil Hospital Israelita Albert Einstein, São Paulo, SP, Brazil.

**Keywords:** Peripheral arterial disease, Chronic limb-threatening ischemia, Atherosclerosis, Angioplasty, Amputation, surgical, Calcium, Lower extremity, Computed tomography, angiography

## Abstract

**Objective:**

To evaluate the association between lower limb artery calcium scores in preoperative computed tomography angiography and outcomes of patients with critical limb ischemia who underwent revascularization procedures in the aortoiliac segment.

**Methods:**

We retrospectively reviewed 14 interventions performed on 11 patients. The calcium score was calculated in segments of the aorta, iliac, femoropopliteal, and below-the-knee arteries. The evaluated outcomes included amputation, patency, death, subsequent revascularization, and readmission. Major adverse limb events were defined as amputation, subsequent revascularization, or readmission. The patients were followed through a 12-month period.

**Results:**

Higher aortic calcium scores were associated with amputation within 30 days (5,767.6 versus 805.3; p=0.02). Amputation during the first year correlated with a higher below-the-knee arteries calcium score (672.4 versus 163.25; p=0.04). Subsequent revascularization within 30 days was associated with a higher aortic calcium score (3,686.8 versus 645.2; p=0.008), while below-the-knee arteries calcium scores were associated with revascularization within 30 days (155.5 versus 817; p=0.05) and 12 months (158.875 versus 679.4; p=0.019). Readmissions within 1 year were more prevalent with higher below-the-knee arteries calcium scores (882.4 versus 32; p=0.011). Major adverse limb events were related to below-the-knee arteries calcium scores at 30 days (158.875 versus 679.4; p=0.019) and 1 year (12.3 versus 910.1; p=0.002).

**Conclusion:**

Below-the-knee arteries calcium score is a predictor of long-term revascularization, amputation, readmission, and major adverse limb events. The aortic calcium score was associated with amputation and subsequent revascularization within the first 30 days.

## In Brief

Calcium scores were evaluated in the lower limbs of patients with clinical limb ischemia after aortoiliac revascularization. The aortic calcium score was related to the short-term outcomes of amputation and subsequent revascularization. Similarly, the calcium score in below-the-knee arteries was associated with revascularization and amputation at 12 months.

## Highlights

■ The calcium score can be calculated using computed tomography angiography.

■ The aortic calcium score was associated with amputation and subsequent revascularization within 30 days.

■ The calcium score in below-the-knee arteries was higher in the patients who underwent subsequent revascularization and amputation at 12 months.

## Graphical abstract

**Figure:**
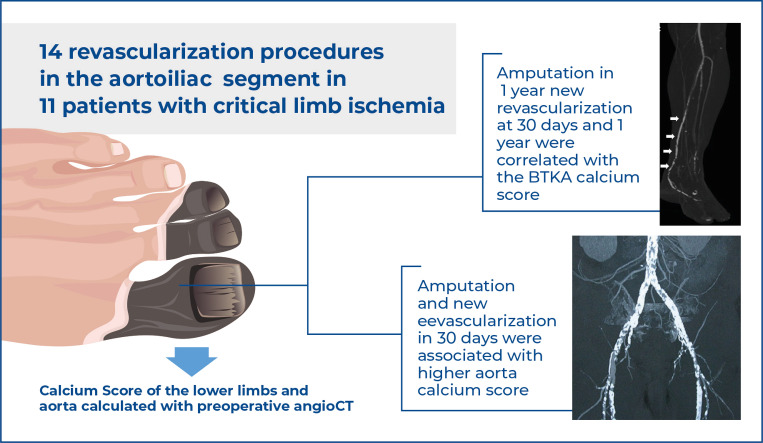

## INTRODUCTION

Vascular calcification (VC) is a common finding in patients with peripheral arterial diseases (PAD). Its incidence increases with age and is directly related to cardiovascular risk factors, such as hypertension, *diabetes mellitus*, and chronic kidney disease.^1^ Several studies on coronary artery calcification (CAC) have shown a direct relationship between the amount of CAC and cardiovascular events. Thus, it is used in clinical practice to stratify the cardiovascular risk in patients with PAD.^2^

Vascular calcification in other arterial beds has also been associated with cardiovascular events and mortality,^3^ and these relationships vary depending on the affected vessel. This suggests that the location and severity of calcifications in different arteries provide unique information on mortality.^4^

Calcification of lower limb arteries (CLLA) has not been as thoroughly explored. There is no standardized methodology for calculating calcium scores (CS) to evaluate CLLA, as different studies use different methods and definitions. However, a direct relationship between CLLA and cardiac events has been reported in patients with PAD.^5^ Higher CLLA levels have been associated with more severe PAD, as patients with ischemic wounds often exhibit higher calcification levels.^6^

The relationship between CLLA and revascularization procedures for PAD has only been evaluated in one study, which focused on patients with critical limb ischemia (CLI) and below-the-knee occlusions treated with endovascular intervention. Using preoperative non-enhanced computed tomography to calculate CS, a lower rate of technical success and a higher rate of amputation were observed in patients with higher CS.^7^

To the best of our knowledge, no prior studies have evaluated the relationship between CS and postoperative outcomes in patients undergoing aortoiliac revascularization.

## OBJECTIVE

To evaluate the association between calcium scores measured by preoperative computed tomography angiography and the outcomes of patients with critical limb ischemia who underwent lower limb revascularization in the aortoiliac segment.

## METHODS

We retrospectively reviewed 14 interventions performed on 11 patients who underwent aortoiliac revascularization for CLI (open and endovascular procedures) using preoperative computed tomography angiography (CTA) between January 2017 and June 2018. The study was approved by the ethics committee of *Hospital Israelita Albert Einstein* (CAAE: 94178818.5.0000.0071; #2953495), and informed consent was obtained from all patients who agreed to participate and were still undergoing follow-up.

Patients were evaluated on four occasions: preoperatively, immediately postoperatively, and 30 days and 1 year after the procedure. Preoperative CTA was used to assess CS. During the three postoperative evaluations, five outcomes and one combined outcome were analyzed.

The five postoperative outcomes studied were: 1) amputation (including major amputation), 2) patency (clinical pulse assessment or imaging with duplex ultrasound), 3) death, 4) subsequent revascularization of the operated limb, and 5) readmission.

The combined outcome studied was major adverse limb events (MALE), defined as any amputation, subsequent revascularization, or readmission.

Computed tomography angiography images were obtained using Toshiba Aquilion One (64 and 160 detectors), Aquilion CXL (128 detectors), or GE Optima 660C (64 detectors), with the same technical parameters for image acquisition. The contrast volume was defined according to patient weight (100–145mL) and administered through an injection pump at 4–6mL/s.

Each preoperative CTA was analyzed to calculate the amount of calcification in different arterial segments. The quantification of the VC by segment was expressed as CS. Preoperative CTA analysis included VC assessment in the following segments: 1) aorta (starting immediately below the lowest renal artery until the iliac bifurcation), 2) iliac (common and external iliac arteries), 3) femoropopliteal (common and superficial femoral arteries and popliteal artery), and 4) below-the-knee arteries (BTKA) (tibiofibular trunk, tibial, and fibular arteries).

In addition to the four segments, the total score of the operated limb (SOL; sum of the scores of the iliac, femoropopliteal, and BTKA of the revascularized limb) and the total calcium score (TCS, total score of all segments in both limbs) were also analyzed.

### Calcium score calculation

The methodology used to calculate CS in the lower limbs was adapted from that of Agatston et al. for CAC scoring, which uses non-contrast computed tomography (CT) images to quantify calcium plaques in the coronary arteries.^8^ However, as the standard protocol for aortic and lower-limb CTA does not include pre-contrast phase acquisition at our institution, we developed our own post-processing methodology for CS calculation. The angiographic phase (phase with contrast) was processed using an Advantage Workstation (AW) 4.6 (GE Medical Systems) to measure the contrast density in Hounsfield Units (HU) within specified regions of interest (ROI).

We chose the ROI as follows: above the abdominal aortic bifurcation, in the popliteal artery at the level of the joint line, and in the anterior tibial artery shortly after its emergence. Each ROI was circular, occupying two-thirds of the vessel diameter and excluding adjacent calcifications. Using the Threshold Tool, structures were selectively defined to retain calcifications while removing contrast based on HU requirements. For each average contrast value of the points of interest, a 30% margin was added to ensure satisfactory removal of the contrast and to maintain vessel calcification. Images were then reformatted on the AW and saved with 2.5mm x 2.5mm thickness, to quantify calcification through the Vitrea FX Workstation (Vital Images, USA) using the Agatston method; the number obtained was the CS.^1^

The ROI above the abdominal aortic bifurcation was used to remove contrast and calculate the CS for the aortic and iliac segments. The ROI of the popliteal artery was used in the same manner as that of the femoropopliteal segment, and the HU measurement of the ROI of the anterior tibial artery was used to calculate the CS of the BTKA segment.

We initially calculated the average CS of the four segments, in addition to the SOL and TCS. Subsequently, we evaluated the relationship between each of the five postoperative outcomes and the combined outcome using the six calcium scores at each of the three postoperative periods.

The average TCS was 5,519. The highest TCS was 17,183 and the lowest was 905.

### Statistical analysis

Qualitative characteristics of the patients were reported using absolute and relative frequencies, and quantitative characteristics were reported using summary measures (mean, standard deviation, median, minimum, and maximum).

The respective CS values were reported according to the outcomes and compared using the Mann-Whitney test. The analysis was performed using SPSS software for Windows (version 22) at 5% significance level.

## RESULTS

Most patients were male (54.5%) and all patients had arterial hypertension. Only 18% of participants had never smoked. The clinical and demographic characteristics of the study participants are shown in table 1.

**Table 1:** Clinical and demographic characteristics of patients included in the study

Characteristics	n (%)
Gender	
Female	6 (54.5)
Male	5 (45.5)
Mean age	63.8
Mean Body Mass Index	23.9
Hypertension	11 (100)
Diabetes	6 (54.5)
Dyslipidemia	4 (36.3)
Smoking history	
Never smoked	2 (18.2)
Previous smoker	4 (36.3)
Current smoker	5 (45.5)
Previous coronary revascularization	2 (18.2)
Coronary insufficiency	1 (9)
End-stage renal disease	1 (9)
Transplantation	1 (9)
Cerebrovascular accident	3 (27.2)
Previous peripheral revascularization	5 (45.5)
Previous amputation	
Major	3 (27.3)
Minor	2 (18.2)
Rutherford Classification	
5	9 (81.9)
4	2 (18.2)
TASC Classification	
A	2 (14.3)
B	5 (35.7)
D	7 (50.0)
Revascularization Technique	
Endovascular	11 (78.5)
Open surgery	3 (21.5)

TASC: Trans-Atlantic Inter-Society Consensus Document on Management of Peripheral Arterial Disease.

Most patients were in the sixth decade of life and had a history of smoking, hypertension, and *diabetes mellitus*. Five patients had undergone prior amputations. All patients had CLI; the majority had severe vascular lesions (TASC D), and most were treated with endovascular intervention (78.5%).

Technical success was achieved for all interventions (open and endovascular) in this series. A total of 10 endovascular revascularization procedures and four open surgeries were performed. Only one clinical complication occurred (postoperative myocardial infarction).

The median CS increased with the severity of TASC classification. The median CS for TASC 1, 2, and 4 were 2,713, 532, and 180, respectively. No patients were classified as TASC 3. However, TASC classification was not associated with any of the analyzed outcomes.

Three minor amputations and one major amputation were performed in the same operation with the revascularization procedure, as previously planned. During the 1-year follow-up, there were two other major and three minor amputations in the first six months.

Four patients required subsequent revascularization on the operated limb within the first 30 days. Three patients had stent occlusion and required subsequent endovascular procedure and one patient had acute thrombosis after open surgery revascularization and was treated with open embolectomy.

The primary patency rate was 85.7% at 30 days and 50% at 1 year. One death occurred during the 1-year follow-up. The CS values measured for each segment are listed in table 2.

**Table 2:** Calcium scores for each arterial segment

	Mean (SD)	Range (Min.–Max.)
Aorta	1,514.2 (2,089.7)	0–8,091
Iliac artery	3,029.6 (7,256.0)	0–22,265
Femoropopliteal artery	902.3 (1,757.6)	0–5,746
BTKA	404.6 (723.4)	0–2,246
Score of the operated limb	4,717.1 (7,958.9)	61–22,420
Total calcium score	5,519.8 (6,942.2)	905–17,183

BTKA: below-the-knee arteries.

Amputation within 30 days was associated with aortic CS (5,767.6 *versus* 805.3; p=0.02); likewise, the occurrence of amputation within 1 year was significantly correlated with the BTKA score (672.4 *versus* 163.25; p=0.04) (Table 3).

**Table 3:** Relationship between calcium score and amputation

	30 days	1 year
Aortic CS		
Non-amputation	805.3	704.4
Amputation	5,767.6	2,971.8
p value	0.022	0.083
BTKA CS		
Non-amputation	359.8	163.25
Amputation	355.5	672.4
p value	0.308	0.045

BTKA: below -the-knee arteries; CS: calcium score.

There was no statistically significant relationship between patency or death with CS in any arterial segment. Subsequent revascularization at 30 days was more prevalent in patients with higher aortic CS (3,686.8 *versus* 645.2; p=0.008), while higher BTKA CS was associated with revascularization at 30 days (155.5 *versus* 817; p=0.05) and 1 year (158.875 *versus* 679.4; p=0.019) (Table 4).

**Table 4:** Relationship between calcium scores and subsequent revascularization

	30 days	1 year
Aortic CS		
Revascularization	645.2	714.9
No revascularization	3,686.8	2,953
p value	0.008	0.147
BTKA CS		
Revascularization	155.5	158.9
No revascularization	817	679.4
p value	0.05	0.019

BTKA: below-the-knee arteries; CS: calcium score.

Readmission within 30 days showed no relationship with CS in any arterial segment. However, readmissions within 1 year showed a statistically significant relationship with BTKA CS (882.4 *versus* 32; p=0.011). Moreover, elevated BTKA CS was associated with MALE at 30 days (158.875 *versus* 679.4; p=0.019) and 1 year (12.3 *versus* 910.1; p=0.002) (Table 5). Aortic CS was also significantly related with the occurrence of MALE within 1 year (522.29 *versus* 2,506.14; p=0.026).

**Table 5:** Relationship between BTKA calcium scores and the occurrence of MALE

BTKA CS	30 days	1 year
MALE	679.4	910.1
Non-MALE	158.875	12.3
p value	0.019	0.002

BTKA: below-the-knee arteries; CS: calcium score; MALE: major adverse limb events.

## DISCUSSION

Vascular calcification is commonly observed in histological studies of different vascular beds and at different stages of atherosclerotic disease.^9^Its development occurs through metabolic and inflammatory processes related to atherosclerotic plaques.^6^ Standardized quantification of VC in coronary arteries is clinical useful for stratifying cardiovascular risk in these patients.^10^

Although there is already a well-established methodology for calculating CAC, a standard method for calculating peripheral CS is not well established, as each study uses its own methodology and definitions.^7,11^

In this study, we evaluated the postoperative outcomes of patients with CLI using preoperative lower-limb CTA. Although non-enhanced imaging was not included, post-image processing techniques allowed intravascular contrast exclusion, enabling the standardized quantification of calcifications.

In the iliac artery, VC correlates with tissue loss in patients with PAD. Higher iliac VC have also been observed in patients with worse left ventricular function.^6^

In 2008, Guzman et al. observed that anterior tibial artery CS, evaluated using non-enhanced CT, was superior to the ankle-brachial index in predicting ischemia progression in patients with PAD.^12^

In our study, we found that BTKA CS is an important predictor of postoperative outcomes. Patients with higher BTKA CS are at a higher risk of amputation 1 year after aortoiliac revascularization surgery. These data suggest that BTKA CS can be used as a marker for preoperative assessment of amputation risk in these patients.

Kang et al. observed that a higher VC in the tibial arteries was associated with lower rate of technical success rates in endovascular interventions and increased amputation risks.^7^

In the present study, BTKA CS showed an association with amputation, readmission, and subsequent revascularization within 1 year, suggesting that it is a good predictor of long-term outcomes. Higher BTKA CS values highlight the need for closer surveillance of these patients. However, unlike Kang’s findings, we were unable to show any relationship between BTKA CS and technical success, probably because of our small sample size.^7^

Interventions in the aortoiliac territory have good long-term patency rates. However, the need for new interventions to maintain patency is common.^13^ In the coronary artery, CS has been identified as a risk factor for restenosis after angioplasty.^14^ The occurrence of re-interventions in our study, however, was not related to any of the calculated CS.

In patients with PAD, aortic CS has been shown to be inversely proportional to the value of the brachial ankle index, suggesting a correlation with the clinical severity.^15^ In our study, aortic CS was significantly associated with early outcomes. Subsequent revascularization and amputation in the first 30 days were more frequent in patients with higher CS in this segment, which may be associated with technical difficulties due to VC in the aortoiliac segment.

This study has several limitations. It is a single-center, retrospective study with no control group and a small sample size. Prospective multicenter studies with a greater number of patients are welcome and may suggest more significant observations.

## CONCLUSION

The calcium score in lower limbs can be quantified in a standardized manner using preoperative computed tomography angiography analysis. Below-the-knee arteries calcium score has been shown to be a predictor of long-term outcomes of subsequent revascularization, amputation, readmission, and major adverse limb events. Aortic calcium score was significantly associated with early outcomes such as amputation and subsequent revascularization within 30 days in patients who underwent revascularization of the aortoiliac segment.
